# Cognitive synaptopathy: synaptic and dendritic spine dysfunction in age-related cognitive disorders

**DOI:** 10.3389/fnagi.2024.1476909

**Published:** 2024-10-03

**Authors:** Francisco J. Barrantes

**Affiliations:** Laboratory of Molecular Neurobiology, Biomedical Research Institute, Pontifical Catholic University of Argentina (UCA), Argentine Scientific and Technological Research Council (CONICET), Buenos Aires, Argentina

**Keywords:** cognition, cognitive impairment, synapse, dendritic spine, memory, ageing, synaptopathies, Alzheimer disease

## Abstract

Cognitive impairment is a leading component of several neurodegenerative and neurodevelopmental diseases, profoundly impacting on the individual, the family, and society at large. Cognitive pathologies are driven by a multiplicity of factors, from genetic mutations and genetic risk factors, neurotransmitter-associated dysfunction, abnormal connectomics at the level of local neuronal circuits and broader brain networks, to environmental influences able to modulate some of the endogenous factors. Otherwise healthy older adults can be expected to experience some degree of mild cognitive impairment, some of which fall into the category of subjective cognitive deficits in clinical practice, while many neurodevelopmental and neurodegenerative diseases course with more profound alterations of cognition, particularly within the spectrum of the dementias. Our knowledge of the underlying neuropathological mechanisms at the root of this ample palette of clinical entities is far from complete. This review looks at current knowledge on synaptic modifications in the context of cognitive function along healthy ageing and cognitive dysfunction in disease, providing insight into differential diagnostic elements in the wide range of synapse alterations, from those associated with the mild cognitive changes of physiological senescence to the more profound abnormalities occurring at advanced clinical stages of dementia. I propose the term “cognitive synaptopathy” to encompass the wide spectrum of synaptic pathologies associated with higher brain function disorders.

## 1 Introduction

Brain function relies on an intricate, multilevel web of anatomical connections among a vast array of neurons and their arborizations, organized in poly-synaptic local and long-range circuits that constitute the neuronal network at large, linking functionally interrelated hubs of neuronal nuclei and larger, macroscopic brain anatomical structures. Current brain-wiring diagrams are addressed by the field of connectomics at the macro- (Sporns et al., 2005), meso- (Zeng, 2018) and micro-scale (Rah et al., 2015). The latter level of organization deals with the synapse, the basic unit of chemical and electrical transmission. One level below, at the molecular scale, connectomics drills down to the building blocks on which interneuronal contacts rely to be able to codify and de-codify electrical and chemical signals-the latter through neurotransmitters and their receptors. These molecules occur in sizeable numbers and are continuously being synthesized, transported, anchored at cell surfaces or organelles, recycled, and destroyed to maintain homeostasis-at a high energy cost. Some of these molecules are common to many neurons, while some confer specificity to selective subsets of neurons. Analogously, the subcellular neuronal structure where information transfer (electrical or chemical) takes place between neurons-the synapse-has both molecular commonality *and* synapse-specific constituents.

Structural and/or functional alterations of the synapse fall under the umbrella of synaptopathies. The term synaptopathy has been readily adopted in relation to hearing and cochlear synapses, with ca. 1,700 references in Pubmed at the writing of this review, vs. ~1,400 references to other synapse alterations unrelated to the former. Recent reviews have described various general properties of synaptopathies in neurological diseases (Lepeta et al., 2016), the influence of inflammatory factors that define immune synaptopathies (Pozzi et al., 2018), and more recently some of the methods currently available to study synapses with a focus on synaptopathies at various levels of resolution (Hindley et al., 2023).

This review describes the most salient characteristics of subtle cognitive decline along physiological ageing and the stadium of objective mild cognitive impairment of the prodromic and preclinical stages of dementias, focalizing on the most common form of these maladies- Alzheimer disease (AD)- and contrasts them with the full psychometrically assessable deterioration and neuronal and synaptic loss at the stage of advanced dementia in several neuropsychiatric diseases. Starting from circuitry connectomics right through to the recently addressable changes in the dynamics of postsynaptic dendritic spines, examples will be drawn from the emerging field of synaptopathies attributable to dysfunction of neurotransmitter systems. The ultimate aim is to find common elements and specific differences to describe what I define as “cognitive synaptopathy,” a concept embracing the ample spectrum of synapse changes from physiological brain senescence, passing through the intermediate mild cognitive impairment (MCI) alterations associated with the lengthy preclinical stages of some neurodegenerative diseases like AD, up to the marked synapse alterations and losses associated with profound cognitive impairment at advanced clinical stages of AD and other dementias. For reasons of space there are a number of topics that this review does not cover, such as the analysis of individual neurotransmitter systems and cholinergic neurotransmission in particular, the physiological and psychological bases of cognitive functions, and the clinical and anatomopathological hallmarks of AD.

## 2 Build-up of cognitive function and dysfunction

### 2.1 Cognitive encryption at the circuitry level

Cognition can be succinctly defined as the higher mental ability to learn, think, and know (Savarimuthu and Ponniah, 2024). Knowledge of the underlying connectomics at several levels of resolution is a prerequisite to comprehending the structural bases of cognitive functions. There is already substantial understanding of structure–function correlations related to higher cognitive functions such as decision-making, cognitive flexibility and goal-directed behavior at the circuitry level, with the prefrontal cortex (PFC) and particularly the medial PFC (mPFC) as a key hub that orchestrates cognitive functions of other brain regions like the hippocampus and the striatum. These cognitive functions are interrelated with reward- and aversion-based learning- drivers of behavior- (Pastor and Medina, 2021) and memory functions related to circuits between the retrosplenial cortex (RSC) and other cortical areas such as the primary visual cortex, linked in turn with higher cognitive areas, e.g., the subicular, cingulate, and midcingulate cortices (de Almeida-Filho et al., 2021). The PFC also modulates the balance between memories that co-localize in the same structure yet compete, giving pre-eminence to declarative and episodic memory in detriment of statistical learning. This is part of the concept of multiple-memory systems (Sherman et al., 2024; Atkinson and Shiffrin, 1968). The reward and punishment system is associated with distributed domains of the PFC cortex: redundant interactions between orbitofrontal and ventromedial PFCs, and a punishment subsystem mediated by connections between the insular and dorsolateral cortices, have recently been identified in this dual learning process (Combrisson et al., 2024).

### 2.2 The hippocampus and its cortical connectivity

**Figure 1:**
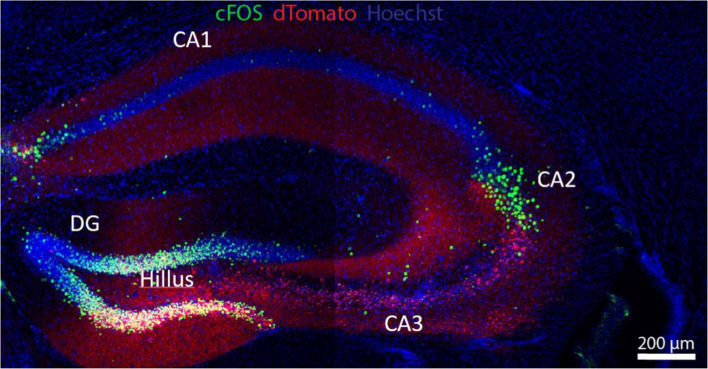
The hippocampus is a key anatomic formation for the encoding of memories, most especially through its interaction with the PFC. The hippocampus receives polysynaptic afferent inputs from the entorhinal cortex ending in the dentate gyrus (DG); DG neurons send mossy fibers to pyramidal cells in CA3, in a well-known example of presynaptic short-term facilitation and plasticity. Axons from the pyramidal cells make synaptic contacts with CA1 neurons via Schaffer collateral pathways. Hippocampal efferents loop back to the PFC and the inferior temporal cortex. This polysynaptic pathway is involved in semantic memory. In addition, a direct cortico-hippocampal pathway intervenes in episodic (recollection of events) and spatial (recognition) memory. The hippocampus was stereotaxically injected with an AAV9: hSyn1-Cre-P2A-dTomato. The red is dTomato filling the infected neurons. The green is Cre recombinase that was immunofluorescent stained with antibody. UV fluorescence (blue) displays nuclei stained with Hoechst stain. Flox transgenic mouse via Cre-loxP system. Micrograph kindly provided by Omar A.A. Shennib, from Dr. MeganWilliams’ Laboratory, University of Utah.

The hippocampus is a key organizer of cognitive functions and a variety of spatial working memory and non-spatial processing mnemonics (Cherubini and Miles, 2015), as epitomized by its spatial representations or “universal metric for space,” i.e., place-selective neurons and their role in navigation (O'Keefe, 1979). The *Cornu ammonis 1* (CA1) receives inputs from the entorhinal cortex- the gateway between the hippocampus and the neocortex- where they either loop back to the entorhinal region in a direct circuit essential for memory formation, or add a detour at the dorsal subiculum before reaching entorhinal cortex layer 5; this polysynaptic circuit is important for rapid updating of episodic memory and retrieval-driven fear responses (Roy et al., 2017). In the context of cognition, the anterior/dorsal hippocampus (CA1 region, Figure 1) has been classically associated with spatial processing and navigation, whereas the posterior/ventral hippocampus (CA2) has been primarily associated with emotional behavior (Fanselow and Dong, 2010), but currently this contention is being replaced by the notion that both anatomical regions are required for spatial learning (Avigan et al., 2020). The ventral hippocampus in general, and the CA1 region in particular, project to emotion and affection-related brain areas like the basolateral amygdala, the nucleus accumbens and the PFC, and are involved in emotional processing and the modulation of stress responses. CA1 dysfunction is thus related to affective disorders (Fanselow and Dong, 2010) and is also one of the first brain areas to show signs of dendritic dystrophy at early stages of tauopathies (Shi et al., 2020).

The hippocampus contains place cells, a subset of neurons that fires when animals move through a specific space in their environment (place field), “remapping” as they move from one space to the next, such that neighboring place cells firing in the first environment do not necessarily remain neighbors in the next. In contrast, entorhinal cortex contains grid cells, a type of place-selective neuron characterized by firing at regular intervals as the animal navigates an open area; grid cells store and integrate information about location, distance, and direction of the environment in grid fields that move and rotate in concert, but they do not remap: the correlation between neighboring grid cells is maintained across environments in stable grid fields (Fyhn et al., 2007). Thus, spatial memory representations in hippocampal place cells work on the basis of multiple, individual environment-specific representations, whereas entorhinal cortex grid cells maintain a constant, universal structure that enables the animal to represent and update position with the same mechanism in all environments. The CA2 region (Figure 1) is key to social behavior. Social interactions induce changes in CA2 place cell electrical activity, and CA2 neurons are involved in the subsequent processing of memory related to such social interactions: the consolidation of spatial memory depends on the reactivation of those place cell neurons that were active during recent behavior (Oliva et al., 2020). The dentate gyrus and discrete cortical regions of CA2 receive input from the hypothalamic supramammillary nucleus, involved with novelty stimuli, e.g., new social encounters; these circuits modulate the processing of social memory (Chen et al., 2020).

The dorsal hippocampus CA3 region (Figure 1) stores the spatial engrams considered to be crucial for integration and retrieval of contextual memory, i.e., connecting a spatial location with an object or reward, exploiting the extensive excitatory recurrent axon collaterals stemming from CA3 pyramidal neurons (Rolls, 2013). Connectivity to the CA3 is also of high functional relevance in cognition: the CA3 fields receive inputs from the entorhinal cortex directly or with a stop at the dentate gyrus via mossy fibers that end in large (5–8 μm) boutons on multi-headed CA3 spines. The CA3 spines configure the so-called “detonator” synapses, with an unusually high number of active zones, enabling a single synapse to trigger the depolarization of CA3 postsynaptic GABAergic inhibitory neurons. The latter pathway performs an important function in memory formation: working as a high-pass filter, it reduces the densely coded messages arising from the entorhinal cortex into a sparse, hippocampal-specific memory encryption (Cherubini and Miles, 2015). The ventral hippocampus is more involved in the processing of the emotional connotations of such memories. Connections between the hippocampus and the RSC coordinate spatiotemporal contextual information and integrate prospective aims in goal-directed behaviors as well as consolidation of emotional memories during sleep (Pronier et al., 2023). Hippocampal circuits undergo both Hebbian and non-Hebbian forms of plasticity during memory encryption [(Çalışkan and Stork, 2018) and see section on plasticity below].

The amygdala, a subcortical limbic structure in the mesiotemporal lobe, is also involved in cognition. The central amygdala is active in learning and encoding of positive, reward-affective information and negative, anxiety and fear behavioral information, awarding valence (positive or negative) and salience (significance) to such stored information. The amygdala-hippocampus-mPFC circuits further process the emotional connotations of such information, in particular fear memory (Genzel et al., 2015) and the lateral habenula regulates the temporal stability of aversive memories and modifies the subjective perception of experiences. Activation of the lateral habenula is required to trigger aversive associative learning (Tomaiuolo et al., 2014).

## 3 Synaptic plasticity, long-term potentiation, and long-term depression

### 3.1 Cognitive encryption at the neuronal cell level

The tracking in real time of higher brain functional information is a needy but technically challenging area, with good progress in some limited instances, such as the remarkable example of current efforts to decipher linguistic engrams. This type of information is stored in individual neurons of the left-lateralized, language-dominant PFC. Williams and colleagues have recently published one of the few available studies, in this case of 10 patients awaiting epilepsy neurosurgery, who listened to auditory input while electrophysiological recordings were obtained from ca. 300 neurons of their PFC (Jamali et al., 2024). The information encrypted in these neurons stems from linguistic input originated in the auditory cortex for speech (or the visual cortex for reading) that converges on the frontal and temporal regions in charge of transducing words into semantic meanings and gathering them into phrase-and sentence-level engrams (Humphries et al., 2006). These word-encoding neurons are organized along the cortical column, which contains the information required for temporally articulating the planning and production of phonetic sequences in the correct order, a requisite for producing coherent speech and dissecting listening input from speaking output (Khanna et al., 2024). The above examples distill decades of research on the functional mapping of distinct and still limited circuits and regions of the brain; for reasons of space, they will not be further expanded on here.

### 3.2 Long-term potentiation (LTP)

The most profound and fastest structural organization of the brain is laid out during the embryonic and postnatal periods. Adult brain retains the capacity to undergo changes in structure and function, especially circuit rewiring in response to sensory and other task-relevant environmental cues and during the continuous process of information storage along the lifespan through a set of diverse and complex mechanisms encompassed under the concept of “plasticity” (Nelson and Turrigiano, 2008; Wefelmeyer et al., 2016; Burrone et al., 2002). The Hebbian theory has provided appealing mechanistic explanations for the processes operating in brain during the imprinting of cognitive phenomena and the resulting adaptation of neurons during learning. Hebb’s insights about the changes occurring during this process (Hebb, 1949) can today be succinctly formulated from a solid neurobiological perspective, supported by decades of experimentation, as the plastic changes in synaptic strength resulting from the repeated and persistent pre-synaptic stimulation of the post-synaptic neuron. The structural correlates, also profusely documented in more than half a century of research on excitatory responses (Nicoll, 2017), are the adult hippocampal neurogenesis at the cellular level (Kempermann et al., 1997; Kempermann, 2019; Gonçalves et al., 2016) and the changes in shape and increase in size of the post-synaptic structure, the dendritic spine (Trachtenberg et al., 2002; Yuste and Bonhoeffer, 2001; Bellot et al., 2014; Segal, 2017; MacGillavry and Hoogenraad, 2015) at the subcellular level. These structural changes, when sustained in time, give rise to an enduring condition, the so-called long-term potentiation (LTP) (Bear and Malenka, 1994; Stevens, 1998; Kroker et al., 2011) (Goda, 2008; Nicoll, 2017; Piette et al., 2023). LTP was first described by Bliss and Lomo (Bliss and Lomo, 1973) and since inception of the concept 50 years ago, has become central to attempts to understand brain cognitive and behavioral physiology and pathophysiology. The pleomorphic character of LTP even at the simplest levels -a single neuron or an elementary circuit-has given rise to the concept of “plasticitome” (McFarlan et al., 2023). LTP has not only emerged as the dominant hypothesis to explain the cellular bases of acquisition, storage, consolidation and retrieval of memory engrams (Bliss and Lomo, 1973; Bliss and Collingridge, 1993; Tsien et al., 1996; Josselyn and Tonegawa, 2020), but its dysfunction is currently conceived in pathophysiological terms as the dysregulated phenomenon common to several synaptopathies present in both neurodegenerative and neurodevelopmental neuropsychiatric diseases.

Synaptic plasticity is classically associated with excitatory synapses, and the paradigmatic excitatory neurotransmitter in brain is glutamate. Glutamatergic synapses undergo activity- and experience-dependent forms of synaptic plasticity- LTP and LTD. For instance, acquisition and formation of danger avoidance in rodents -a learned conditioned response- is initiated by activation of different glutamatergic receptors [AMPARs, metabotropic and particularly NMDARs (Izquierdo et al., 2006)]. Receptor activation generates molecular modifications in the neurons of the CA1 region of the hippocampus that resemble those involved in CA1 LTP. Activation of AMPARs leads to their trafficking along the plane of the membrane (Kasai et al., 2010) and their transient immobilization at the PSD, where they are anchored by the scaffolding protein PSD-95, which is present in most excitatory synapses, adjuvates in the distribution of neurotransmitter receptors, and awards stability to the spine. This process has been described as “input-specific” (Ehlers et al., 2007). The glutamatergic axo-dendritic synapse, typically a single axon *en passant* bouton that innervates a glutamatergic site, has in general the characteristic mushroom-type spine morphology. The scaffolding proteins PSD-95 and SHANK (SH3 and multiple ankyrin repeat domains) are distinctive inhabitants of the glutamatergic synapse, stabilizing and interfacing glutamatergic receptors (GluRs) with the cytoskeleton (Hering and Sheng, 2001; Sala et al., 2001) at the PSD. PSD-95 and SHANK belong to the family of PDZ domain (structural domain of 80–90 amino-acids found in the signaling proteins)-containing scaffold proteins. The postsynaptic expression of SHANK potentiates presynaptic function, a strong indication of the important role played by the SHANK scaffold protein in synaptic plasticity.

**Figure 2:**
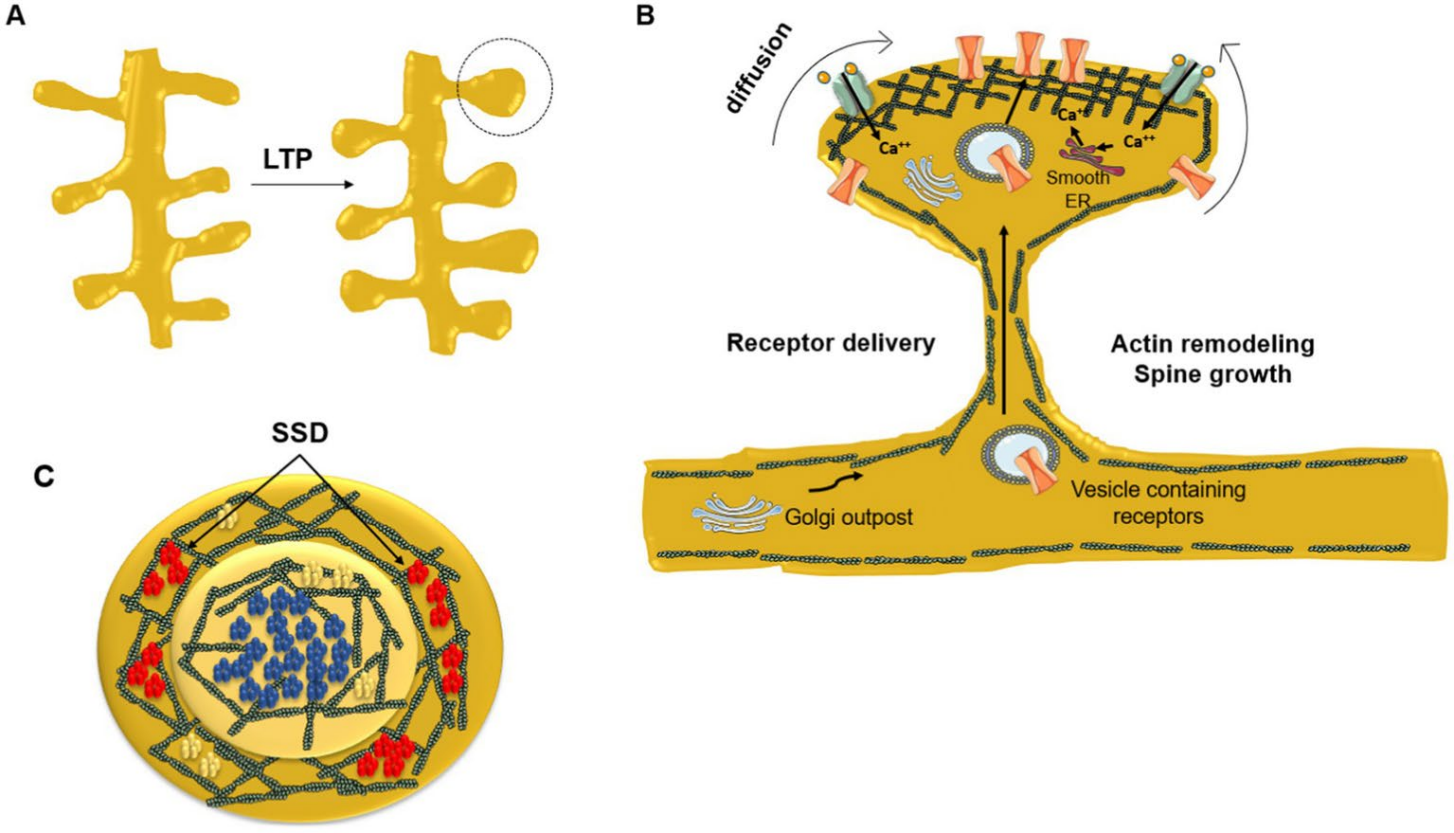
Dynamic redistribution of neurotransmitter receptors during synaptic plastic changes. **(A)** Enlargement of spine head due to long-term potentiation (LTP). **(B)** Remodeling of the submembrane actin meshwork plays a key role in the mechanical restructuration of the dendritic spine driven by the repeated stimulation of the synapse. Newly synthesized receptors and receptors residing in extrasynaptic areas diffuse laterally toward the PSD. The neurotransmitter receptor number increases at the PSD central area directly facing the presynaptic neurotransmitter release active zone. At the crest of the spine receptors are corralled and immobilized by the filamentous actin submembrane network and by scaffolding proteins like SHANK and PSD-95. Dendritic Golgi outposts contribute to posttranslational modification of newly synthesized receptors delivered through the spine neck to the PSD via vesicular transport. **(C)** End-on view of the PSD. NMDARs (blue) occupy the center of the PSD in a single nanocluster, whereas AMPARs (red) are usually located peripherally in the form of smaller nanodomains (sub-synaptic domains, SSD) surrounding the central, single NMDAR nanodomain. In contrast, mGluR5 (yellow) are aggregated into small clusters or homogeneously distributed at the PSD. Modified from Vallés and Barrantes (2021b), under Creative Commons Attribution (CC BY) license (https://creativecommons.org/licenses/by/4.0/).

Transient trapping of neurotransmitter receptors in the active zone of the synapse occurs when the diffusional exchange of receptors between synaptic and extrasynaptic regions is low. AMPAR immobilization is triggered by phosphorylation; stabilization of AMPARs at the PSD increases synaptic efficacy (via LTP), whereas removal of these receptors from the PSD leads to long-term depression (LTD, see next subsection). The lateral positioning of AMPARs within the PSD is important because their alignment with presynaptic glutamate release sites has a strong influence on the probability of receptor activation (MacGillavry et al., 2011). Figure 2 provides a schematic depiction of the distribution of NMDA-subtype glutamatergic receptors at an excitatory synapse. The correlation between receptor topography at the center of the PSD and gain of synaptic efficacy is clearly apparent.

Twenty percent of spines in the mature brain lack the PSD-95 protein and are short-lived; this minority pool of spines constitutes a highly dynamic and mostly transient population, frequently rewiring and remodeling, and probably not involved in long-lasting circuits (Berry and Nedivi, 2017). Changes at the single synapse level can be further expanded into changes in connectivity of multiple synapses and scaled up into plastic modifications of micro-and macro-circuits at the meso-and macro-scale in the brain. The plastic behavior comes at a price: highly plastic systems are unstable (Nelson and Turrigiano, 2008).

The Hebbian basic functional coupling -or enhanced connectivity-between two neurons is predominant in, though not exclusive to excitatory synapses: GABAergic and glycinergic inhibitory synapses can be dynamically regulated through similar processes (Petrini et al., 2014; Barberis and Bacci, 2015; Pennacchietti et al., 2017). There are far fewer GABAergic neurons than glutamatergic neurons, but they display a great variety of subtypes. GABAA receptors (GABAARs) overwhelmingly mediate inhibitory neurotransmission in the CNS. Synaptic ionotropic GABAARs mediate fast and transient responses, while extrasynaptic GABARs are involved in tonic inhibition. Nicotinic acetylcholine receptors (nAChRs) also participate in synaptic plastic phenomena. They are ubiquitously distributed in brain and contribute together with the glutamatergic system to the modulation of LTP (Toricelli et al., 2021). In addition, nAChRs are involved in the mechanisms of dendritogenesis and the morphological remodeling of spines and generation of new spines, i.e., spinogenesis, in the hippocampus (Toricelli et al., 2021; Jackson et al., 2019) and other key brain regions associated with cognition (Borroni and Barrantes, 2021). Various theoretical models [the Sotelo model, the Miller/Peters model, and the filopodial model, reviewed in (Lai et al., 2016)] provide different accounts of spinogenesis. Activation of nAChRs induces LTP and inhibits the induction of LTD, thus positively contributing to the processes of learning and memory (Pastor and Medina, 2023). This is at the root of the early compromise of the cholinergic system at initial stages of cognitive dysfunction and is one of the hallmarks of cognitive impairment in AD (Babic, 1999).

Besides the increased connectivity and the recruitment of neurotransmitter receptors, another characteristic feature of LTP is the enlargement of the postsynaptic compartment, most conspicuously observed in pyramidal neurons, and its counterpart shrinkage in LTD. However, imaging of spine volume for long period using 2-photon microscopy has disclosed volumetric changes under normal synaptic transmission, even after blocking synaptic activity, a phenomenon dubbed “intrinsic fluctuation” of dendritic spines (Yasumatsu et al., 2008). These authors developed a mathematical model based on the theory of random fluctuations in an attempt to understand the long-term persistence of large volume spines beyond thin ones, and the rates of generation and elimination, as well as steady-state stability of these structures. Spines with larger heads are more stable and are apparently needed for long-term memory formation, whilst smaller and thinner spines are more unstable, easily eliminated and possibly responsible for the initial steps of memory acquisition (Gipson and Olive, 2017).

A central conundrum of learning and memory phenomenology is the timing of synaptic changes relative to the cognitive experience (Gipson and Olive, 2017). An inherent notion of classical Hebbian plasticity is that cognitive experiences are converted into memories through input-specific synaptic plastic modifications at the time of learning. However, this concept is challenged by views that consider synaptic plasticity to be neither strictly input specific nor restricted to the specific time window of its induction. Using a weak associative conditioning protocol, where optogenetic stimulation of sensory thalamic input to the amygdala was paired with a foot shock, no memory formation was observed. However, if the same input was potentiated minutes before or even a day later, the experience-shock pairing was converted into a lasting memory, suggesting that (a) conversion of a transient experience into a lasting memory is not restricted to the time of the experience, and (b) the switch is not exclusively related to the synapses that participated in the initial triggering event, and can be influenced by past and future events (Faress et al., 2024).

Several dendritic spine mechanisms have been postulated to account for the changes occurring during plastic phenomena associated with learning and memory beyond the increase in the number or volume/size of spines. Among such mechanisms are the formation of multiple spines between a single axon and a dendrite (Toni et al., 1999), the transient expansion of existing spines during the course of LTP in hippocampal synapses (Lang et al., 2004), or the stabilization of existing spines and strengthening of synapses at the onset of behavioral learning (Roberts et al., 2010). Clusters of dendritic spines has been invoked as an information storage mechanism operating in cortical circuits. Using an episodic-like learning paradigm and 2-photon optical microscopy to follow spine dynamics in retrosplenial cortex *in vivo*, Silva and coworkers found that hotspots of high spine turnover before learning drives future loci and rates of spine clustering and learning and memory outcomes (Frank et al., 2018). The subjacent molecular mechanisms invoke the stabilization of the F-actin network in the excitatory glutamatergic spine (Bellot et al., 2014) and its induction by protein kinase D (Bencsik et al., 2015), or cholinergic triggering of IP3 receptor activation in hippocampal CA1 pyramidal neurons (Fernandez et al., 2008). Co-active clusters of 3 to 6 synaptic inputs are observed along the dendrites of layer 2/3 pyramidal neurons in the mouse primary visual cortex well before eye opening; these clusters display a non-random distribution, occurring in distinct domains that may reflect discrete computational modules along the dendrites (Leighton et al., 2024).

If the LTP dogma is a valid construct to explain long-term memories, and the synapse provides the structural/molecular machinery to this end, one of the most fascinating questions in Neurobiology is how the encoded memories are maintained over such prologued periods, even throughout an individual’s entire lifespan. This is particularly intriguing in view of the half-life of most proteins involved in the memory-related phenomena at the synapse (Basu and Lamprecht, 2018). Recent observations may shed light on this enigma. A key molecule involved in LTP, namely Ca^2+^/calmodulin kinase II (CaMKII), catalyzes the phosphotransferase enzymatic reaction of its own molecule (autophosphorylation) and of several downstream target proteins. It has recently been reported that the autophosphorylation reaction coupled to CaMKII binding to the GluN2B subunit of the glutamate receptor is required to initiate LTP and maintain it, i.e., to sustain synaptic memory (Chen et al., 2024). Another interesting hypothesis involving CaMKII proposes that memories survive the turnover of synaptic molecular constituents because this kinase regains its activity from preexisting CaMKII molecules present at the synapse (Lee et al., 2022).

### 3.3 Decreased synaptic strength in long-term depression (LTD)

Long-term depression (LTD), the counterpart of LTP, is the activity-dependent diminution of synaptic strength associated with either a decrease in presynaptic neurotransmitter release or a reduction of postsynaptic receptors, also observed with NMDA, AMPA or kainate rapid excitatory glutamatergic transmission, or slow metabotropic glutamate-mediated transmission. Imaging dendritic spines in acute hippocampal slices from neonatal rats with two-photon time-lapse microscopy showed that the LTD induction by low-frequency stimulation leads to a noticeable reduction in the size of spines. High-frequency stimulation can reverse the LTD-induced changes and induce LTP. The diminution of spine size involves activation of the NMDA subtype of excitatory glutamatergic receptors and calcineurin, following a mechanism akin to that operative for LTP, but spine shrinkage is mediated by cofilin and not by protein phosphatase 1, which is operative in LTD, indicating that different pathways operate in the two phenomena (Zhou et al., 2004). Both LTP and LTD play important functional roles, not only in cognitive processes like learning and memory but also during neurodevelopment, and the alteration of the synaptic weight operated through this balance has been discussed within the frame of various neurodevelopmental and neurodegenerative diseases (Walsh et al., 2002; Chen et al., 2006; Zhang et al., 2018). In addition to these relatively long-term phenomena, their shorter-lived counterparts, i.e., short-term potentiation and short-term depression, are also active in synaptic plasticity (Silva et al., 1997; Taschenberger et al., 2016).

## 4 Ageing and cognitive decline along physiological ageing

Ageing is a multidimensional, multisystem complex process occurring at varying rates and intensities of decline in physiological integrity across individuals, and whose precise commencement along the lifespan is virtually impossible to ascertain. Genomic instability, telomere attrition, chronic inflammation, loss of proteostasis (the homeostatic equilibrium of a functional cellular proteome), dysfunctional autophagy, and stem cell exhaustion have been recently listed as hallmarks of cellular ageing, among other factors (López-Otín et al., 2023). A recent proteomics study of *ca.* 6,000 human blood plasma protein samples has revealed that 20% of the population shows strongly accelerated ageing in a single organ, and 1.7% are multi-organ agers, conferring 20–50% higher mortality risk (Oh et al., 2023). Moreover, those individuals with accelerated heart ageing also exhibited accelerated brain ageing. Another proteomics study analyzed 2,897 blood plasma proteins in more than 45,000 individuals in the UK Biobank [a statistically stronger sample, 30 times larger than that in (Oh et al., 2023)] as predictors of major disease morbidity and mortality. The analysis identified 204 proteins showing correlation with other age markers like telomere length or frailty index, and accurately predicted chronological age and the incidence of 18 major chronic diseases (Argentieri et al., 2024). A very recent brain-clock study focused on demographic, socioeconomic and other disparities and inequalities across Latin American and non-Latin American population datasets of healthy patients and those with mild cognitive impairment. The study showed that individuals in Latin American countries had larger brain-age gaps, which the authors attribute to the burden of non-communicable and communicable diseases, socioeconomic inequality, and increased air pollution among other factors (Moguilner et al., 2024). Because of their application to large available databases of populations from diverse geographies, ethnicity and genetic background, these “proteomic-clock” studies that quantitatively evaluate similarities and dissimilarities between biological and chronological brain age will probably play a major role in defining risk factors in neurodegenerative maladies and other age-related diseases.

Cognitive decline is the most distinct sign of brain ageing, and on a par with general ageing, is a heterogeneous phenomenon affecting different individuals in a dissimilar manner, and also dissimilarly compromising different cognitive domains, such as sensory perception memory, attention, executive function, language, reasoning, and space navigation ability (Yang et al., 2023). Physiological brain ageing, as is the case with various other organs and tissues of the economy, entails cellular senescence of both neuronal and glial cells, which accumulate DNA damage. Cellular senescence also involves the arrest of the cell’s ability to divide, together with increases in cell secretory activity, a condition termed senescence-associated secretory phenotype, abnormal or loss of proteostasis and autophagy, accumulation of lipid droplets, DNA methylation and histone post-translational modifications (Sikora et al., 2021). Microglial immunosenescence can parallel the analogous phenomenon in bone marrow hematopoietic stem and progenitor cells (Zhang et al., 2024).

Several of the above ageing-associated changes can in turn trigger low-level chronic neuroinflammation, associated with changes in brain volume that current imaging techniques in clinical practice can reveal. On this basis, neuroimaging-based “brain ageing” has been proposed as a biomarker of brain health status and cognitive ageing (Cole and Franke, 2017) and functional magnetic resonance imaging (fMRI) techniques seconded by deep learning -a form of machine learning- are increasingly being applied to profile more accurately the signatures of brain ageing (Bashyam et al., 2020; Beck et al., 2024). The difference between the actual chronological age and the image-based measure of “brain ageing” is referred to as the above-mentioned brain-age gap estimate, a positive gap being considered a reflection of accelerated cognitive decline (Elliott et al., 2021). Akin to what is observed in engineering and biological systems, functional redundancy of circuits along the lifespan (Sadiq et al., 2021) and neural network redundancy in the brains of higher species are also considered cognitive neuroprotective factors (Glassman, 1987) that mitigate the effects of age on cognition. Intense levels of physical activity in middle-age have been correlated with a decreased risk of cognitive decline in late adult life (Morgan et al., 2012; Sofi et al., 2011; Dik et al., 2003; Frank et al., 2023) A recent meta-analysis of the effects of physical activity on cognition comprising 104 studies and more than 300,000 older adults showed a weak association between physical activity with late-life better cognitive abilities. Despite the lack of strong correlation the authors highlighted the importance of the finding from a population health perspective (Iso-Markku et al., 2024).

### 4.1 Neuronal vulnerability, neuronal loss, and adult neurogenesis

Neurogenesis in the adult human brain is mainly associated with active stem cell niches in distinct areas of the hippocampus and the PFC. Hippocampal adult neurogenesis largely originates in stem cells in the subgranular zone of the dentate gyrus (DG), giving rise to differentiated granule neurons (Denoth-Lippuner and Jessberger, 2021). Since granule neurons together with hippocampal pyramidal cells comprise a brain region particularly enriched in excitatory neurons, it is not surprising that adult hippocampal neurogenesis is considered the paradigm of plasticity at the cellular level, and the constitutive generation of new granule neuronal cells the key to maintaining healthy learning and memory processes. However, adult neurogenesis may not suffice to compensate for the loss of neuronal cell bodies and more importantly of synaptic transmission along ageing. The medial temporal lobe and PFC, loci of learning and cognitive functions, are particularly vulnerable during normal ageing (Burke and Barnes, 2006). The same applies to the hippocampus and the DG in particular (Lazarov et al., 2016; Lepousez et al., 2015) or the PFC (Morrison and Baxter, 2012; Morrison and Hof, 1997), where reversal learning and pattern separation are two typical vulnerable cognitive functions. In their analysis of cerebral cortex synapses along physiological ageing, Morrison and Baxter argue that cognitive impairments do not result from the neuronal loss in the forebrain but from subtle specific changes in hippocampal and PFC synapses (Morrison and Baxter, 2012). Early and selective, region-specific losses of synapses in cerebral cortex and striatum have been reported to result from the concerted action of microglia and the complement cascade in neurodegenerative diseases like Huntington disease (Wilton et al., 2023). Greenwood has maintained the hypothesis that cognitive ageing implies not only loss, but also adaptation to loss, the latter consisting of functional reorganization of brain regions through changes in brain processing strategies (Greenwood, 2007).

Some anatomical changes associated with senescence in brain correlate with changes in cognitive performance, but the heterogeneity of the former precludes a full comprehension of the underlying mechanisms. Amyloid *β* (Aβ) deposition in the neocortex has been reported in necropsy studies of apparently healthy elderly individuals (Morris et al., 1996). Cytoarchitectonically, the DG, the *Cornu ammonis* sectors 1–4, and the subiculum, have been reported to exhibit differential vulnerability to ageing and/or age-dependent susceptibility to neurodegenerative injury affecting cognitive performance (Homayouni et al., 2023). Hippocampal dentate gyrus granule cell losses predominantly derive in dysfunction of declarative memory, whereas those of the PFC are associated mainly with working memory (the cognitive function to hold a relatively small amount of information “in mind”), but according to some authors neuronal death figures are quantitatively minor in cortex and hippocampus (Morrison and Baxter, 2012; Morrison and Hof, 1997). Reduction in the complexity and amount of dendritic arborization have been reported to occur in human cortex with increasing age (Scheibel et al., 1975).

### 4.2 Genes, cognition and longevity

Model animal studies have revealed that mutations in some genes are associated with longer lifespans, most notably in *C. elegans*, a nematode with more than 800 genes related to ageing. Mammals, including humans, also possess genes that affect the lifespan (Hook et al., 2018), prominent among these are the genes of the insulin (Park et al., 2021; Kwon et al., 2010) and the mTOR (Green et al., 2022; Tabibzadeh, 2021; Mota-Martorell et al., 2022) pathways. Numerous studies report the effect of calorie restriction as a longevity-favoring factor (Fontana and Partridge, 2015; Hwangbo et al., 2020; Green et al., 2022). Genome-wide association studies (GWAS) have identified more than ten longevity loci in humans, including *APOE* and *5q33.3* (Zeng et al., 2016). Single-nucleotide polymorphisms of the gene coding for the major histocompatibility complex class 2, *HLA-DQB1,* have been associated with lipid homeostasis and long-lived Chinese individuals (Yang et al., 2017).

Epidemiological studies suggest that lifestyle habits may help or be detrimental to cognition, influencing the proclivity toward a healthy cognitive status or inclining the balance toward cognitive decline, respectively (Evans et al., 1989; Reitz and Mayeux, 2014). GWAS have disclosed genetic variants associated with cognitive performance, accounting for up to 10% of the variance in cognitive test performance in adults (Tsapanou et al., 2023). According to this study, genetic predisposition appears to have a stronger influence on cognitive performance in the young and in midlife adults than in older and elderly adults. This contention is supported by other studies indicating that cognitive ability increases significantly, in a linear fashion, from 41% in childhood, to 55% in adolescence, and 66% in young adulthood, as analyzed in a sample of 11,000 pairs of twins from four countries (Haworth et al., 2010). Collectively, these GWAS indicate that cognitive performance is, at least for certain specific cognitive values (Procopio et al., 2022), related to genomic DNA, i.e., heritable. Further studies are needed to fully understand this important aspect of complex traits that converge on normal and dysfunctional cognition. Being a carrier of the *ApoEε4* allele coding for the ApoE4 lipoprotein increases the likelihood of cognitive decline for elderly people (Dubal and Yokoyama, 2020).

While most studies on AD focus on postsynaptic alteration of LTP, impairment of excitatory synaptic transmission and pathological aberrations of dendritic spines, i.e., postsynaptic synaptopathies, recent work is drawing attention to genetic and non-genetic alterations of presynaptic components. Frontotemporal dementia and amyotrophic lateral sclerosis are two pathologies that target genes coding for presynaptic proteins and are possibly affected at the early stages of disease, before synapse loss and neuronal degeneration, and which can lead to profound cognitive synaptopathies (Clayton et al., 2024). Similarly, genes involved in presynaptic functions are mutated or constitute risk factors in autism spectrum disorder (ASD) (Vallés and Barrantes, 2021a; Yeo et al., 2022). The cognitive synaptopathy associated with AD can also affect presynaptic functions, including those mediated by the amyloid precursor protein (APP) and the presenilin paralogs PS1 and PS2, genetically linked to the EOAD variant of AD. Barthet and Mulle highlight three main presynaptic mechanisms possibly affected in the AD synaptopathy: (i) APP fragments binding to presynaptic receptors (e.g., nAChRs and GABA_B_Rs), (ii) presenilins controlling Ca^2+^ homeostasis and Ca^2+^-sensors and (iii) tau regulating the localization of synaptic vesicles and presynaptic molecules (Barthet and Mulle, 2020).

### 4.3 Cognitive performance in the elderly adult and subjective cognitive decline

While autobiographical, emotional and implicit memory, semantic memory and short-term memory appear to be relatively well conserved across the lifespan of healthy individuals and only decline at advanced age, behavioral studies have often documented the waning of the ability of elderly adults to form new episodic memories, to rapidly process information, or to invoke executive processes (Hedden and Gabrieli, 2004), configuring what has been termed normal cognitive ageing (Harada et al., 2013). These forms of memory decay appear to correlate with subtle alterations in both gray and white matter. At the macroscopic neuroanatomical level, volumetric changes in gray and white matter can be recorded in imaging studies, e.g., in the PFC of subjects with mild memory impairment, where loci of strategic episodic encoding and executive processes reside, and in the mass of anterior white matter. Hippocampal volume decline is less conspicuous during normal ageing, although deterioration of functional activity in the hippocampus and cortical connections have been observed in healthy older adults.

Population-based studies report that between 50 and 80% of older individuals (70 years or older) who objectively perform normally in cognitive psychometric tests, report some form of self-perceived decline in cognitive function (Jessen et al., 2010; Ávila-Villanueva et al., 2016; Buckley et al., 2015). This form of cognitive decline is increasingly being observed in clinical practice; individuals refer a deterioration in one or more cognitive domains, e.g., in their mnemonic functions, learning capacity, or ability to engage in cognitive tasks that were perceived as normal in the past, or combinations thereof. This form of decline can be encompassed under the term subjective cognitive decline (SCD). The distinctive feature of this form of cognitive decline is that cognitive performance as judged by comprehensive neuropsychiatric/psychometric tests appears not to be compromised in these individuals, nor is their day-to-day functioning affected. Several clinical criteria for this entity were defined by the SCD-initiative (Jessen et al., 2014; Ávila-Villanueva et al., 2016). The key unifying criterion is a self-experienced and persistent deterioration of cognition relative to a previously perceived normal cognitive status. Objective differential diagnosis between SCD and the MCI present at pre-symptomatic and prodromal stages of neurodegenerative diseases like Alzheimer or Parkinson diseases becomes imperative. The importance of SCD is its (still uncertain) character as a risk factor for mild cognitive impairment (van Harten et al., 2018) or its conversion to dementia (Stogmann et al., 2016). A thorough review of criteria to diagnostically categorize SCD, its degree of severity, possible causes, and specific cognitive areas affected can be found in Jessen et al., (2020). A recent fMRI study found parietal-occipital multifocal impairments that correlated positively with executive functions in SCD individuals, and suggested further use of imaging studies as biomarkers of SCD (Huang et al., 2024).

Do specific changes in dendritic spines accompany physiological brain senescence or do the relatively mild losses of cognition merely reflect a quantitative reduction in the number of dendritic spines or synapses in cognitive-related circuits in normal ageing? At the subsynaptic and molecular level little is known about synaptic structural alterations along normal ageing (Dickstein et al., 2013). Morphological studies of young and aged rhesus monkey pyramidal neurons and synapse density in layer III of area 46 (dorsolateral PFC) revealed a clear age-related loss of dendritic spines (33%) on pyramidal cells and a 32% decreased density of axospinous synapses in the old, cognitively compromised monkeys. The volume of the spine head was found to be the strongest correlate of cognitive behavior (Dumitriu et al., 2010). Reduction in spine density, and in particular of thin spines along lifespan, has been reported to occur in non-human primate PFC. The average head diameter of the thin, non-stubby spines was again suggested to be a better predictor of cognitive status (in this case of recognition memory) than chronological age in the rhesus monkey model (Young et al., 2014). In contrast, Aβ protein deposits are not only observed in AD patients but also in brains of cognitively normal older individuals (Russo et al., 1998; Baker-Nigh et al., 2015).

An intriguing topic worthy of further research is the above-average performance of some cognitively intact oldest-old elderly individuals, a minority subpopulation whose genetic dotation or some other factor(s) may have a protective or mitigating effect against both mortality and cognitive decline (Schmeidler et al., 2019), the so-called protected survivor model. According to this posit, an example of Simpson’s paradox, the association of the risk factor with survival does not change within an individual, but the association in the surviving population changes as its age increases (Silverman and Schmeidler, 2018).

## 5 Alzheimer disease and its diverse and mutating definitions

AD has been considered either a heterogeneous and complex nosological entity with unknown, multifactorial etiology, constituting the most common form of dementia worldwide or, contrastingly, a diffuse clinical syndrome reflecting the gradual accumulation of multiple pathologies (a “continuum”) stemming from risk factors in the course of the lifespan (Richards and Brayne, 2010). Along these lines, a so-called “biological” perspective of AD, convened by the U.S. National Institute of Aging and formulated by the Alzheimer’s Association Workgroup, relies on the underlying abnormal, pathological findings: Aβ deposition, pathological tau protein, and neurodegeneration (Jack et al., 2018). A recent update of this proposal put forward by the same sources reinforces the diagnostic and staging criteria of AD based solely on the neuropathological findings and their biomarkers (see Section 6 below), dispensing with the clinical signs and symptoms (Jack et al., 2024). It is difficult to understand why these neuropathological findings are considered “biological,” and why cerebral amyloidosis defines AD when many healthy individuals with amyloid burden do not develop the disease.

There are two variants of the disease. The genetic form is a dominant familial or autosomal disease; it represents only 1–5% of the total number of cases. Affected individuals are middle-aged, 65 years old or younger, and the clinical entity is referred to as early onset AD (EOAD). The familial variant presents highly penetrant genetic mutations in presenilin 1 (PSEN1), presenilin 2 (PSEN2), or the amyloid precursor protein (APP) (Reitz et al., 2023). The original description of AD by Alois Alzheimer at a psychiatry meeting in 1906 characterized this disease as a neurological-psychiatric entity with a defined anatomopathological correlate ((Alzheimer, 1906) and see (Hippius and Neundörfer, 2003) and (Bermejo-Pareja and del Ser, 2024) for historical perspectives).

The much more frequent sporadic form of AD represents 95% of cases and is dubbed as late-onset (LOAD), occurring in individuals older than 65 years of age. Today, sporadic AD is currently considered a heterogenous, multifactorial disorder characterized by progressive impairment in cognition, memory, emotion, and language in the elderly (Gong et al., 2018). Its pathognomonic necropsy findings are the extracellular Aβ plaques and intracellular tau protein neurofibrillary tangles, consisting of supramolecular aggregates of these proteins in various abnormal conformations (Goedert et al., 2023; Scheres et al., 2023). These pathophysiological hallmarks of pathological brain parenchyma are accompanied by amyloid vasculopathy, consisting of amyloid deposits in brain blood vessels, loss of neuronal cells, signs of neuroinflammation, and reactive astrogliosis. Brain vascular disease, with its impact on the integrity of the blood–brain barrier and its frequent combination with neuroinflammation, accelerates the course of AD [the “nun study” (Snowdon et al., 1997; Snowdon, 2003)] and adds to the cognitive dysfunction of the patient due to its inherent vascular cognitive impairment (Rosenberg et al., 2015). The complex nature of the sporadic form of AD implies that its etiopathogenesis may obey several different mechanisms and pathological pathways across individuals, and even in a single individual (Gong et al., 2018). The roots of one of the most characteristic signs, memory dysfunction at early stages of AD, has been a subject of research for many years, particularly the puzzling aspect of whether it is due to disrupted encoding and consolidation of episodic information, or to impaired retrieval of stored memory engrams. Tonegawa and coworkers showed that in a transgenic mouse model of early AD, activation of hippocampal memory engram cells retrieves memory engrams despite the fact that these mice have memory deficits in long-term memory tests, indicating that it is the retrieval mechanisms rather than memory storage mechanisms that fail. Before amyloid plaque deposition, age dependence correlates with the progressive reduction in spine density of hippocampal dentate gyrus engram cells (Roy et al., 2016).

### 5.1 Toward identifying specific synaptopathy markers in Alzheimer disease

MCI has been defined as the construct intervening between normal ageing and very early stages of dementia, a notion that reached consensus at the Key Symposium in 2003 (Petersen, 2004). Despite the inherent difficulties in assessing with absolute rigor this neuro-psychological entity and the absence of MCI-specific biomarkers (Albert et al., 2011; Alexiou et al., 2017; Ávila-Villanueva et al., 2016), and at variance with dementia at advanced stages of AD, the patient affected by MCI preserves both daily functioning and independence (Petersen et al., 2014).

Several risk factors have been identified in sporadic AD; age stands as the main one (EADB-MR Collaboration, 2023; Corder et al., 1993), followed by being a carrier of the *ApoEε4* gene (Bertrand et al., 1995), However, *ApoEε4* carriers may not develop dementia in their lifetime, and longitudinal studies suggest that this variant of the gene may lead to age-dependent cognitive impairment independently of AD pathology (Palmer et al., 2023). Heterozygosity, i.e., carrying only one copy of *ApoEε4* rises the risk of developing AD by 3-fold, while homozygous carriers with two copies of the gene increase their risk by more than 12-fold. Carrying the allele reduces the age of onset of LOAD, and reduces survival of both LOAD and EOAD (Dubal and Yokoyama, 2020). Hypercholesterolemia, sex, lifestyle, and diet complement the list of main risk factors.

A recent clinical/pathology study on more than 13,000 individuals showed that by age 65 nearly all had abnormal amyloid levels and 75% positive amyloid scans, thus indicating full AD biology and concluding that ApoEε4 homozygotes represent a new genetic form of AD (Fortea et al., 2024).

One of the histopathological hallmarks of AD- the accumulation of Aβ in the extracellular space- gave rise to the amyloid cascade hypothesis (Hardy and Higgins, 1992), a construct that has dominated the field of AD for decades. The hypothesis poses that deposition of the Aβ peptide is the primary cause of the disease, a premise that has not found experimental proof in animal models nor validation through drug or monoclonal antibody therapies addressing Aβ aggregation in clinical practice (Kametani and Hasegawa, 2018). Given its extensive literature cover this subject will not be developed further here.

In terms of specific molecules associated with synaptic plasticity, the myokine/adipokine protein irisin is another candidate biomarker. Fibronectin type III domain-containing protein 5 (FNDC5), is a membrane-bound protein expressed in the hippocampus. Physical exercise results in the cleavage of FNDC5 and the release of irisin. The levels of irisin are diminished in the hippocampus of AD mouse models and knockdown of brain FNDC5/irisin impairs LTP, while FNDC5/irisin increased levels rescue synaptic plasticity and memory in these animals (Lourenco et al., 2019).

### 5.2 Synapse hyperexcitability and tauopathic dendritic degeneration as early synaptopathy signatures of AD

Extracellular low-n oligomers of the tau protein have been reported to cause selective synaptotoxicity (Kaniyappan et al., 2017). Hyperphosphorylated tau induces cognitive decline, dendritic spine loss and mitochondrial abnormalities in a mouse AD model (Kandimalla et al., 2018) and hyperphosphorylated tau is emerging as a likelier candidate of AD initiation and progression than Aβ. Which regions of the brain are likely to develop the first signs of tauopathy? The search for neurodegenerative-susceptible brain areas dates back several decades, and the spread of pathological tau in a “prion-like” fashion from one neuron to the next has been hypothesized to correlate with AD (DeVos et al., 2018) and form the basis of Braak staging of AD-related changes (Braak and Braak, 1991) that can be investigated by tau-PET imaging (Biel et al., 2021).

Loss of synapses is considered the most reliable measurable correlate of cognitive decline in postmortem AD brain parenchyma (DeKosky and Scheff, 1990; Terry et al., 1991). A marked (32%) reduction of layer II entorhinal cortex neurons was reported in necropsies of patients with very mild clinical cognitive dysfunction, at early stages of AD at their time of death (Gómez-Isla et al., 1996). A subset of neurons in layer II of the entorhinal cortex expresses the neurodevelopmental-associated glycoprotein reelin, which is involved in synaptic plasticity in the adult brain (Long et al., 2020). Intracellular Aβ has been observed in these reelin-positive neurons at pre-plaque deposition early stages of AD (Kobro-Flatmoen et al., 2016). Reelin-expressing neurons in layer II are selectively lost in aged monkeys with memory deficits (Long et al., 2020). Advanced stages of AD neuronal loss may amount to more than 15% of cortical neurons, providing one of the strongest neurobiological markers of objective cognitive impairment (Skaper et al., 2017). The lateral entorhinal cortex has emerged as the key seed area for AD early synaptopathy; cell death can amount to 60% in mild AD, rising to 90% in demented patients and, importantly, preceded by excitatory-inhibitory synaptic disbalance resulting in synaptic hyperexcitability, attributable to the diminution of parvalbumin interneurons in animal models (Petrache et al., 2019). Using optical superresolution (STED), structural degeneration of the dendrites in CA1 hippocampal pyramidal neurons has been correlated with the early synaptic hyperexcitability sign in an animal model of AD (Šišková et al., 2014). A recent optical superresolution study using expansion microscopy showed tau oligomers in synapses, and tau oligomer–tagged synapses engulfed by microglia, the CNS-resident macrophages, and also by astrocytes in necropsy tissue of AD patients, with substantial loss of presynaptic (43%) and postsynaptic (38%) structures (Taddei et al., 2023). Microglia secrete the complement protein C1q, a key participant in complement-mediated synapse elimination in physiological neurodevelopment and disease models; interestingly, brain C1q augments significantly through ageing (Scott-Hewitt et al., 2024). Another recent study found that early hyperexcitability of the entorhinal cortex may be attributed to the vulnerability of parvalbumin interneurons, specific to human (and not animal) APP protein, and absent from somatosensory cortex (Goettemoeller et al., 2024). Pyramidal synaptic hyperexcitability and dendritic dystrophy thus appear to be promising pathognomonic signatures of early stages of AD-type tauopathy, preceding tangle and plaque formation (Shi et al., 2020; Goettemoeller et al., 2024). Summarizing, the entorhinal cortex, a key supplier of excitatory sensory inputs to the hippocampus, and the recipient of CA1 and subiculum inputs, is consolidating its involvement as the early, if not the earliest, locus of neuronal injury in AD.

## 6 The search for diagnostic biomarkers of cognitive impairment and AD

The Alzheimer’s Association (AA) recently updated the 2018 criteria for diagnosis of AD (Jack et al., 2018) and established additional ones for the staging of cognitive impairment (Jack et al., 2024). Two groups of biomarkers are defined: early-changing core 1 biomarkers [amyloid positron emission tomography (PET)], cerebrospinal fluid biomarkers, and plasma biomarkers like phosphorylated tau 217, which reflect either the Aβ proteinopathy or the AD phosphorylated tau proteinopathy pathway. Biofluid analyte biomarkers like microtubule-binding region tau-243 and tau-PET constitute the second, later-changing core 2 biomarkers (Jack et al., 2024). An abnormal core 1 biomarker suffices to establish a positive diagnosis of AD, while core 2 biomarkers offer prognostic information. The key issue of this approach is that the two sets of biomarkers are considered sufficient to diagnose AD without recourse to clinical data. There is no universal acceptance of the biomarker-only diagnostic criteria; some patients treated with anti-Aβ monoclonal antibodies are free of cerebral amyloid, yet they still exhibit cognitive impairment (Zhou et al., 2024). Furthermore, according to the AA criteria 20% of all individuals older than ~50 years of age would suffer AD (Gustavsson et al., 2023). Asymptomatic biomarker-positive individuals should be considered at risk of AD and not having AD (Zhou et al., 2024). Having said this, the knowledge acquired in recent years on more specific AD biomarkers, and in particular on imaging and blood plasma biomarkers, is opening new perspectives for presymptomatic diagnosis of AD, as analyzed below.

### 6.1 Positron emission tomography imaging

Based on the notion that glucose hypometabolism is a reflection of the high-energy consumption by brain synapses, PET imaging of glucose hypometabolism with fluorine 18–labeled fluorodeoxyglucose (^18^F-FDG) has been used as an unspecific biomarker of synaptic loss in AD (Rocher et al., 2003). However, until recently there has been no *in vivo* method to specifically examine synapses in the human brain. Using the presynaptic vesicle glycoprotein 2A in the form of a novel PET radioligand, ^11^C-UCB-J, this biomarker was used to quantify synaptic density in the living human brain (Finnema et al., 2016) and to compare amyloid-positive patients exhibiting either MCI or AD dementia with amyloid-negative aged-matched controls (Chen et al., 2018). Patients with MCI and AD dementia had significantly reduced ^11^C-UCB-J in the medial temporal lobe and hippocampus (~40%) that could be related to poorer memory performance. Aβ PET has also been used as a biomarker of AD, but its frustrating aspect is that positivity may precede the onset of clinical symptoms by up to 20 years (Jansen, 2015), and a large percentage of amyloid-positive individuals will not evidence signs of cognitive impairment during their lifetime (Hansson et al., 2017; Hansson, 2021). According to some authors, blood plasma analysis of Aβ appears to be a more reliable alternative to Aβ PET (Janelidze et al., 2016; Li et al., 2022). Though less employed in clinical practice, another imaging modality, tau PET, appears to be more strongly associated with future evolution toward clinical cognitive impairment (Biel et al., 2021). The entorhinal cortex sends outputs to the hippocampal DG, and this in turn connects to the CA3 and CA1 regions: this relatively simple circuit is involved in learning and memory. Although postmortem studies of several cortical areas including entorhinal and transentorhinal cortices show synaptic loss that can be identified in electron microscopy studies, conventional optical microscopy counting of synaptic spots fails to detect such decreases in neocortex (Domínguez-Álvaro et al., 2019). A possible explanation for this discrepancy is that autopsy studies are overwhelmingly conducted on individuals in terminal phases of disease, while synaptic loss could be restricted more to earlier stages, or even prodromic/presymptomatic phases.

### 6.2 Blood plasma biomarkers

Blood plasma tests are gaining momentum as biomarkers of AD. Their advantage over other diagnostic approaches is evident: they avoid the invasiveness and risks of cerebrospinal fluid tests based on Aβ42/40 ratiometric measurements and their cost is substantially lower than imaging techniques like PET scans. According to some authors P-tau181, which quantifies phosphorylated tau protein at amino acid threonine 181 in human blood plasma, constitutes the most reliable biomarker of future evolution of AD (Janelidze et al., 2020; Karikari et al., 2020). The rationale behind the p-tau 181 test is that it correlates in most cases with neurofibrillary tangle pathology. Elevated p-tau 181 levels and neurofilament light chain protein have also been associated with the emergence of psychotic symptoms like hallucinations and delusions in a cohort of 752 AD patients with MCI, apparently with predictive value of accelerated cognitive and functional decline (Gomar and Koppel, 2024). Plasmatic tau protein phosphorylated at amino acid threonine 217 has recently been reported to be a biomarker of individuals with subthreshold levels of Aβ accumulation (Janelidze et al., 2024), with an accuracy comparable to that of cerebrospinal fluid biomarkers (Ashton et al., 2024). The recently reported organ ageing signature in a blood plasma study also appears to show promise (Oh et al., 2023). Functional ultrasound is another imaging technique used to study the mesoscopic organization and functional dynamics of the brain with improved spatial resolution over fMRI (Macé et al., 2011). Given their participation in the ageing-associated processes listed in the section on Ageing and Cognitive Decline, the possible use of non-coding RNAs, in particular circular RNAs as biomarkers of AD, has been suggested [see review in (Canoy et al., 2024)].

### 6.3 Other molecular markers of cognitive impairment and neuropathological changes

The Tg2576 rodent model of AD overexpresses a mutated human amyloid precursor protein (APPswe). At the circuit level, the animals exhibit a selective loss of midbrain DAergic neurons in the ventral tegmental area (VTA) at pre-plaque stages, although substantia nigra pars compacta DAergic neurons are not affected (Nobili et al., 2017). VTA DAergic neuronal loss reduces the DA outflow in the hippocampus and nucleus accumbens (NAc) shell.

Severe cognitive impairment is an objective sign, amenable to clinical diagnosis as a common dysfunction in various forms of dementia. This sign is generally associated with advanced stages of AD; the condition can be described as a cognitive deficit that impairs daily functioning, quality of life, and often loss of independence of the patient. A related concept, “cognitive frailty” can be defined as the concurrent presence of physical frailty and cognitive impairment without coexisting dementia. As with dementia, the underlying etiopathogenic mechanisms of cognitive frailty are still uncertain; a wide palette of considerations like sociodemographic factors, social status, nutritional status, geriatric syndrome, physical and cognitive activities, comorbidities, medications, gut-derived metabolites and structural changes in the brain have been implicated (Sugimoto et al., 2022).

Kalirin-7 is a brain specific guanine-nucleotide exchange factor for the small GTPase Rac1, indirectly participating in dendritic spine morphogenesis, maintenance, and plasticity (Penzes and Jones, 2008). As discussed in the section on synaptic plasticity, Rac1 is involved in the regulation of spine morphology via actin dynamics. Activation of NMDARs in pyramidal neurons results in CaMKII-dependent phosphorylation of kalirin-7, which in turn activates Rac1, producing fast size increases of existing spines (Xie et al., 2007). Kalirin-7 also interacts with AMPA receptors and controls their synaptic expression. Thus, kalirin-7 expression and spine localization are required for activity-dependent spine enlargement and enhancement of AMPAR-mediated synaptic transmission. Soluble Aβ oligomer binding to mature hippocampal neurons induces the depletion of kalirin-7 from spines, while kalirin-7 overexpression prevents Aβ oligomer-induced spine degeneration (Xie et al., 2019), suggesting its possible relevance in AD.

## 7 Nanoscale neurobiological alterations in synaptopathies: focus on dendritic spines

### 7.1 Dendritic spines

In addition to the synaptic inputs received directly on their soma, neurons have a second, more extensive surface in the form of arborizations serving as the key input or receptive field of the cell: the dendritic tree. These ramifications of the neuronal cell collect and integrate signals arising from many (10^2^–10^3^) other neurons. The receptive field is further amplified by small protrusions along the dendrites. Structurally, these predominantly mushroom-shaped protrusions of about 1–2 μm in diameter -the dendritic spines- are connected to the main shaft of the dendrite by a thin neck (Figure 2). Dendritic spines were discovered by Ramón y Cajal (1888) based on observations of Purkinje cells in bird brains that he published in Spanish in the first issue of the journal that he launched and financed [see historical accounts by DeFelipe (2015); Garcia-Lopez et al. (2007); Heck and Santos (2023); Segal (2002); Yuste and Bonhoeffer (2001)]. Remarkably, on the sole basis of their morphology and location, Ramón y Cajal hypothesized that these structures, which he coined spines, could be involved in cognition. A formidable inference that was initially received with skepticism but subsequently became one of the bases of the neuronal theory.

From a functional viewpoint, dendritic spines are the main (though not exclusive) unitary postsynaptic element of excitatory synapses in the adult mammalian brain, specialized in decoding the incoming chemical signal from a single axon. A further specialized structure of the dendritic spine is the post-synaptic density (PSD), an electron-dense zone of the postsynaptic compartment discovered during the early days of electron microscopy in the Neurosciences (De Robertis and Bennett, 1954; Kennedy, 1998). This is a vulnerable synaptic structure in AD and other neurodegenerative dementias (Gong and Lippa, 2010). Most spines exhibit a single, continuous PSD, though some spines possess discontinuous or “perforated” PSDs.

In the course of ontogenetic development, the dendritic spine changes its morphology -both shape and size- over variable timescales, from a small protrusion (“stubby” type), passing through the thick “mushroom” morphology prior to adopting their “adult” subcellular anatomy characterized by a thin neck and a spherical head. The proteome of the dendritic spine is complex and includes neurotransmitter receptors, receptor anchoring and scaffolding proteins, a dynamic cytoskeleton, motor proteins, and a variety of enzymes catalyzing multiple reactions in the tiny postsynaptic compartment (Vallés and Barrantes, 2022).

One of the relatively recent innovations in structural Neurobiology is the increasing application of various types of microscopies to image the synapse in health and disease with unprecedented spatiotemporal resolution. Maximal spatial resolution is still dominated by solid-state biophysical techniques like structural NMR, electron microscopy (EM) and, increasingly, by cryo-EM. Due to the high energies required for X-ray and electron interrogation of biological structures, fixed specimens are almost inevitably required for biological samples. Yet the information obtained in recent years on the structure of Aβ and tau protein is remarkable. For instance, until recently the *in situ* atomic structure of Aβ in the human brain was unknown. Employing cryo-fluorescence microscopy-targeted cryo-sectioning, cryo-focused ion beam-scanning EM, and cryo-EM, the architectures of Aβ and tau pathology in the brain of a postmortem AD could be resolved. Aβ plaques contained a mixture of linear and branched fibrils and protofilaments, arranged in parallel arrays and lattice-like structures, whereas tau deposits consisted of parallel clusters of linear, unbranched filaments (Gilbert et al., 2024).

Complementing the information obtained at the atomic scale by the above techniques, knowledge of the nanoscale organization of the synapse has been strongly impacted by the advent of superresolution optical microscopies (“nanoscopies”) (Moerner, 2015; Hell, 2015; Betzig, 2015) and their applications in Neurobiology (Willig and Barrantes, 2014; Eggeling et al., 2013; Choquet et al., 2021; Choquet and Hosy, 2020; Choquet and Triller, 2013). Nanoscopy is opening the possibility of interrogating brain connectomics at the nanoscale, borrowing from the serial sectioning and volumetric (3D) reconstruction techniques developed for electron microscopy (EM), but using fluorescence imaging alone as readout or in combination with EM (Micheva et al., 2010b; Rah et al., 2015; Drawitsch et al., 2018) or spatially localized transcriptomics (Minehart and Speer, 2021; Galindo et al., 2021; Micheva et al., 2010a). Complementing the new optics-based nanoscopies, expansion microscopy combines the isotropic swelling of the biological tissue embedded in a swellable polymer and imaging in a conventional wide-field microscope (Chen et al., 2015; Wassie et al., 2019).

### 7.2 New structural details stemming from optical superresolution microscopy

Initial applications of the new techniques aimed at exploiting the enhanced spatial resolution afforded by optical nanoscopy: the study of the distribution of peripheral neurotransmitter receptors at the cell surface (Kellner et al., 2007) and morphological plastic changes in central synapses (Groc et al., 2007; Giannone et al., 2010; Borgdorff and Choquet, 2002; Choquet and Hosy, 2020). Stochastic optical reconstruction microscopy (STORM), a form of single-molecule superresolution microscopy, revealed the multiple localizations of CaMKII in spines, where it exhibits slow translational motion, and at points quite distant from the synapse (Lu et al., 2014). NMDAR activation was found to prompt the immobilization of CAMKII at the two types of sites.

A recent application of stimulated emission depletion (STED) superresolution microscopy to a murine model of schizophrenia (the Tcf4tg mouse) has revealed increased numbers of immature spines in the PFC of young adult animals (Badowska et al., 2020). Steffens et al. also looked for spine alterations in the SOD transgenic mouse, which models the neurodegenerative disease amyotrophic lateral sclerosis, characterized by loss of motor neurons. Prior to the stage of neuronal degeneration, examination of the layer V cortical neurons revealed a marked diminution in spine density on the neuronal apical tufts, with an apparently compensatory increase in spine head size in the residual stable spines (Steffens et al., 2021), reinforcing the view that ALS is a characteristic synaptopathy (Fogarty, 2019).

### 7.3 Real-time observation of live synapses and dendritic spines

It is however the improved temporal resolution of the new optical nanoscopies that has introduced a game-changing experimental paradigm into the interrogation of synaptic structure by revealing nanoscale changes in the dynamics of the synapse, gaining access to these subcellular structures through imaging modalities applicable to live tissue and even live animals. The group of Eric Betzig at Janelia Farm perfected the use of one of the less detrimental optical superresolution microscopies, structured illumination microscopy (SIM), to image live zebrafish larvae and mouse brain with a 2-fold resolution improvement over the diffraction limit at about 10 frames per second (Turcotte et al., 2019).

**Figure 3:**
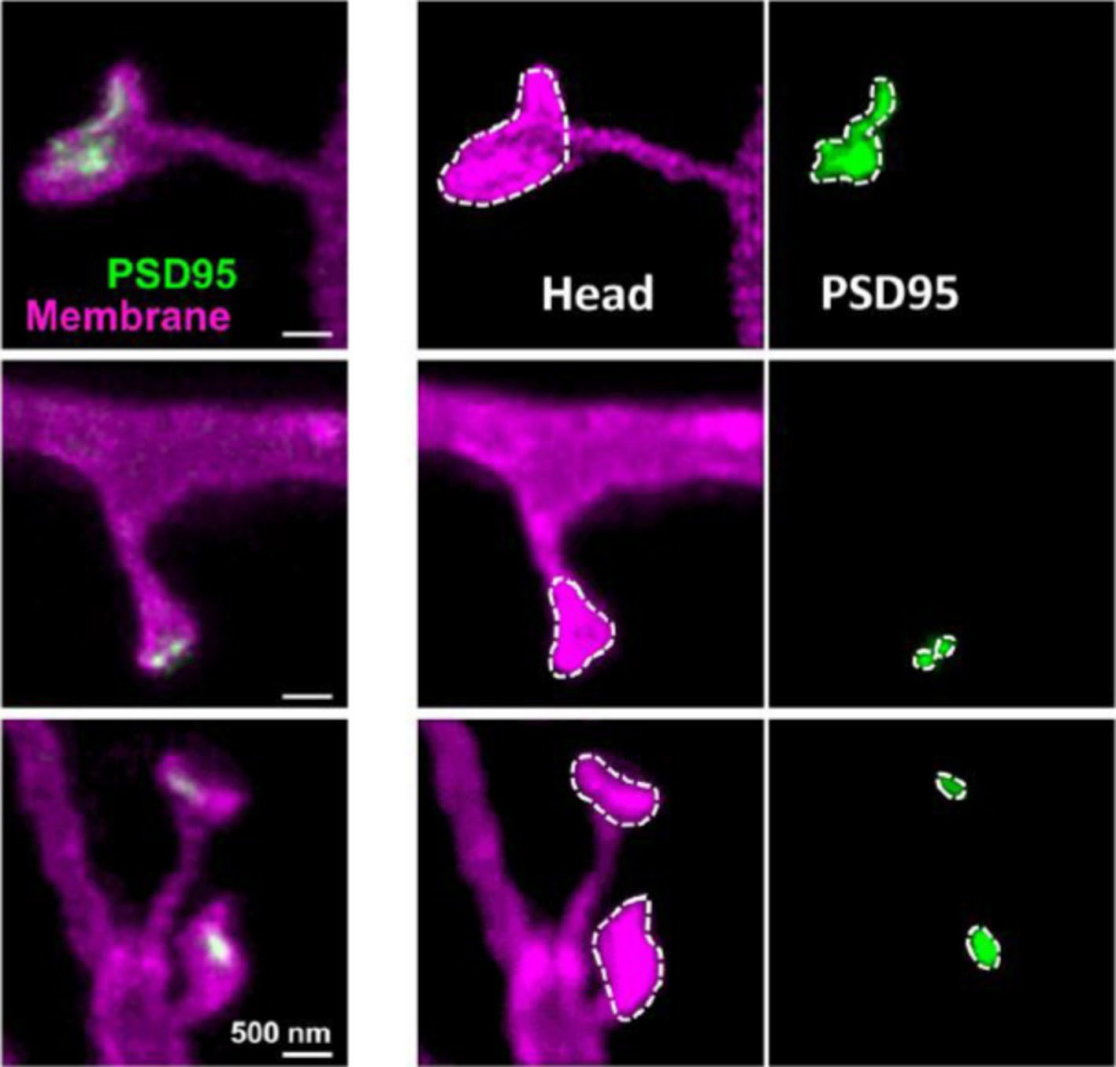
Stimulated emission depletion (STED) images showing larger dendritic spines (magenta) and associated PSD-95 protein nanoaggregate areas (green) in mice housed in environmentally-enriched cages as compared to those of animals reared in standard cages. The middle column shows the spine heads; the right column the PSD-95 assemblies outlined in white dashed lines, and the left column the merged images. Reproduced from Wegner et al. (2022), under Creative Commons Attribution (CC BY) license (https://creativecommons.org/licenses/by/4.0/).

It has long been known that dendritic spines exhibit rapid motility, changing their shape in seconds. The shape changes involve a remodeling of the spine actin cytoskeleton and actin-based protrusive activity from the spine head (Hering and Sheng, 2001). Capitalizing on the experience gained a decade ago in Stefan Hell’s laboratory in imaging static snapshots of dendritic spines in live neurons in culture and in a live rodent brain (Berning et al., 2012; Willig et al., 2014), Katrin Willig and her group have recently followed dendritic spine morphology for hours (Wegner et al., 2018) and up to one month at a resolution of 100 nm in the cortex of a transgenic mouse (Steffens et al., 2021) with STED microscopy. More recently, combining nanoscopy imaging with environmental enrichment paradigms in a mouse model, they could follow synaptic plastic changes in live animals (Wegner et al., 2022). To this end they used STED superresolution microscopy to image the scaffolding protein PSD-95 in live mice in a cortical region known to undergo plastic changes upon visual stimulation (Greifzu et al., 2014; Baroncelli et al., 2010; Kalogeraki et al., 2017). Willig and coworkers analyzed the spine morphology and found that large spine heads tended to shrink, and smaller spine heads to grow following environmental enrichment, thus converging toward a more uniform end-point size distribution than in control mice (Wegner et al., 2022). The nanoscale assembly size of the scaffolding protein PSD-95 in the PSD was temporally uncorrelated with the increase in the spine head size in the animals subjected to environmental enrichment stimulation (Figure 3).

In the Steffens et al. study, persistent spines were found to have almost three times larger heads than transient spines. They also possessed a 35% longer and 25% thicker neck compared with transient spines. Thin spines with filopodia-type morphology (~2 μm long and 0.2 μm in diameter) had average lifetimes shorter than 3–4 days (Steffens et al., 2021). Another recent study addressed the PSD-95 protein using MINFLUX, one of the most powerful optical nanoscopy modalities, to investigate its molecular and supramolecular organization (Morris et al., 2023). A major population of PSD-95-containing super-complexes consisted of two copies of PSD-95, 12.7 nm apart. Interestingly, each PSD-95 subunit was sequentially replaced in days/weeks, a process that varied across different brain regions. Subunit replacement was slowest in the cortex, where the lifetime of the PSD-95 protein is longest. That proteins within the PSD can be maintained by gradual replacement of individual subunits provides a mechanism for the maintenance of their stable and yet dynamic organization. Furthermore, the experimental data supports Francis Crick’s “simplest” hypothesis on memory, which posited that “*molecules in the synapse interact in such a way that they can be replaced by new material, one at a time, without altering the overall state of the structure*” (Crick, 1984). The finding of PSD proteins with long lifetimes strongly suggests a correlation with long-term memory storage; their differential anatomical distribution in brain and their preferential enrichment in superficial layers of the cortex (Morris et al., 2023), where long-term memories are purportedly stored, adds support to this correlation. One of the appealing aspects of this construct is that long-term memories would reside in the lifetime of tangible molecules, susceptible of experimental verification. Precisely on these grounds, the verifiability of working memory [originally termed short-term memory or “short-term store” in the modal or multi-store model of Atkinson and Shiffrin (1968)], conceptualized as the link between perception and long-term memory (Baddeley, 2012), is experimentally more demanding.

Proteins are encoded by more than 17,000 genes in a human cell, and individual members of this large proteome display unique spatial relationships, which contribute to and, in some contexts, dictate function (Lycas and Manley, 2024). Current developments in superresolution optical microscopy are providing new insights into the spatial arrangement of proteins in the synapse and helping to determine their abundance and relative positions at the molecular scale. Furthermore, nanoscopy imaging provides direct dynamic evidence of dendritic spine remodeling and its correlation with cognition in animal models subjected to learning paradigms. This new evidence is offering structural insight into synaptic plasticity in live subjects, in some cases following the changes in dendritic spines for prolonged periods, thus providing glimpses into the realms of learning and long-term memory cognitive processes.

## 8 Conclusions and future prospects

The complexity of physiological brain senescence and of neurodegenerative diseases like AD, and the shifting paradigms and therapeutic failures associated with the latter make fully apparent our incomplete understanding of these subjects and the need of further research. Progress is being made in the use of more sensitive and specific biomarkers and blood plasma proteomic analysis as predictors of pathology and chronological age (“proteomic-clock” studies), two areas that promise accelerated developments.

In terms of cognitive synaptopathy, spatially-resolved proteomics of individual synapses with concomitant knowledge of their precise neuronal origin is currently at hand; expanding this procedure across the constellation of synapses converging on a given neuronal subtype is more demanding and time-consuming, but needed to gain a better understanding of cognition-related (and other) brain circuits at the synaptic (learning and adaptation plastic changes) and molecular levels and their differential susceptibility to synaptopathies. The inception of superresolution nanoscopies in the field is likely to provide new insights into the structural bases of synaptic plasticity and cognitive functions at large.

We still cannot unambiguously assert whether reduced synaptic number and/or density precedes, occurs simultaneously with, or downstream of the Aβ and/or tau pathologies. Unraveling the mechanisms associated with memory encoding and its long-term storage and retrieval is another needy area of research. Are there neuron type-specific molecules associated with these processes? Can the recent molecule replacement experiments be verified for different types of memories? Could these findings lead to more reliable biomarkers for the preclinical stages of AD and other neurodegenerative diseases? These and other similarly unresolved questions make synaptopathies a fascinating field in the Neurosciences.
