# Correction: Cognitive synaptopathy: synaptic and dendritic spine dysfunction in age-related cognitive disorders

**DOI:** 10.3389/fnagi.2026.1845297

**Published:** 2026-06-18

**Authors:** Francisco J. Barrantes

**Affiliations:** Laboratory of Molecular Neurobiology, Biomedical Research Institute, Pontifical Catholic University of Argentina (UCA), Argentine Scientific and Technological Research Council (CONICET), Buenos Aires, Argentina

**Keywords:** cognition, cognitive impairment, synapse, dendritic spine, memory, ageing, synaptopathies, Alzheimer disease

In the published article, the caption of **Figure 1** was erroneously incomplete and incorrect. The sentences: “The neuronal soma in green corresponds to enhanced green fluorescent protein (eGFP) driven by a C-fos promoter. Red staining corresponds to Cre-P2A-dTomato fluorescent protein from Discosoma sp., introduced by cerebroventricular injection with an adeno-associated virus (AAV)” should have been: “The hippocampus was stereotaxically injected with an AAV9: hSyn1-Cre-P2A-dTomato. The red is dTomato filling the infected neurons. The green is Cre recombinase that was immunofluorescent stained with antibody.”

The corrected caption of **Figure 1** appears below.

“**Figure 1**. The hippocampus is a key anatomic formation for the encoding of memories, most especially through its interaction with the PFC. The hippocampus receives polysynaptic afferent inputs from the entorhinal cortex ending in the dentate gyrus (DG); DG neurons send mossy fibers to pyramidal cells in CA3, in a well-known example of presynaptic short-term facilitation and plasticity. Axons from the pyramidal cells make synaptic contacts with CA1 neurons via Schaffer collateral pathways. Hippocampal efferents loop back to the PFC and the inferior temporal cortex. This polysynaptic pathway is involved in semantic memory. In addition, a direct cortico-hippocampal pathway intervenes in episodic (recollection of events) and spatial (recognition) memory. The hippocampus was stereotaxically injected with an AAV9: hSyn1-Cre-P2A-dTomato. The red is dTomato filling the infected neurons. The green is Cre recombinase that was immunofluorescent stained with antibody. UV fluorescence (blue) displays nuclei stained with Hoechst stain. Flox transgenic mouse via Cre-loxP system. Micrograph kindly provided by Omar A.A. Shennib, from Dr. Megan Williams' Laboratory, University of Utah.”

The original version of this article has been updated.

